# P-1435. Effectiveness of RSV Immunization among Infants in Their First RSV Season in the United States, 2023-2024 and 2024-2025 Seasons

**DOI:** 10.1093/ofid/ofaf695.1622

**Published:** 2026-01-11

**Authors:** Amanda B Payne, Steph Battan-Wraith, Sarah E Reese, Cassandra A Hathaway, Sara Y Tartof, Karthik Natarajan, Stephanie Irving, Shaun J Grannis, Sean M Chickery, Gabriela Vazquez-Benitez, Kristin K Dascomb, S Bianca Salas, Cassandra Bezi, Lina S Sy, Bruno Lewin, Melissa S Stockwell, Ashley B Stephens, Jungmi Han, Allison L Naleway, Brian E Dixon, Colin Rogerson, Thomas Duszynski, William F Fadel, Sarah W Ball, Jingran Cao, Charlene E McEvoy, Tamara Sheffield, Daniel Bride, Julie Arndorfer, Joshua Van Otterloo, Amber Kautz, Allison Avrich Ciesla, Morgan Najdowski, Josephine Mak, Jennifer DeCuir, Ryan E Wiegand, Ruth Link-Gelles

**Affiliations:** CDC, Atlanta, Georgia; Westat, Rockville, Maryland; Westat, Rockville, Maryland; Westat, Rockville, Maryland; Kaiser Permanente Southern California, Pasedena, CA; Columbia University, New York, New York; Kaiser Permanente Center for Health Research, Portland, Oregon; Indiana University, Indianapolis, Indiana; Westat, Rockville, Maryland; HealthPartners Institute, bloomington, Minnesota; Intermountain Healthcare, Murray, Utah; Kaiser Permanente Southern California, Pasedena, CA; Kaiser Permanente Southern California, Pasedena, CA; Kaiser Permanente Southern California, Pasedena, CA; Kaiser Permanente Department of Research and Evaluation, Pasadena, CA; Columbia University Irving Medical Center, New York, New York; Columbia University Irving Medical Center, New York, New York; Columbia University, New York, New York; Kaiser Permanente Center for Health Research, Portland, Oregon; Regenstrief Institute, Indianapolis, Indiana; Regenstrief Institute, Indianapolis, Indiana; Regenstrief Institute, Indianapolis, Indiana; Regenstrief Institute, Indianapolis, Indiana; Westat, Rockville, Maryland; HealthPartners Institute, bloomington, Minnesota; HealthPartners Institute, bloomington, Minnesota; IntermountainHealth, Salt Lake City, Utah; Intermountain Healthcare, Murray, Utah; Intermountain Healthcare, Murray, Utah; IntermountainHealth, Salt Lake City, Utah; Centers for Disease Control and Prevention, Atlanta, Georgia; Centers for Disease Control and Prevention, Atlanta, Georgia; CDC, Atlanta, Georgia; Division of Healthcare Quality Promotion, Centers for Disease Control and Prevention, Atlanta, Georgia; Centers for Disease Control and Prevention, Atlanta, Georgia; Centers for Disease Control and Prevention, Atlanta, Georgia; Centers for Disease Control and Prevention, Atlanta, Georgia

## Abstract

**Background:**

During the 2023-2024 and 2024-2025 respiratory virus seasons, two products, a maternal vaccine (Abrysvo, by Pfizer) and an infant monoclonal antibody (nirsevimab), were recommended in the United States to protect infants against severe respiratory syncytial virus (RSV) disease during their first RSV season. We estimated product effectiveness (PE) against RSV-associated emergency department (ED) encounters and hospitalizations among infants in their first RSV season during these seasons.

**Table 1.**
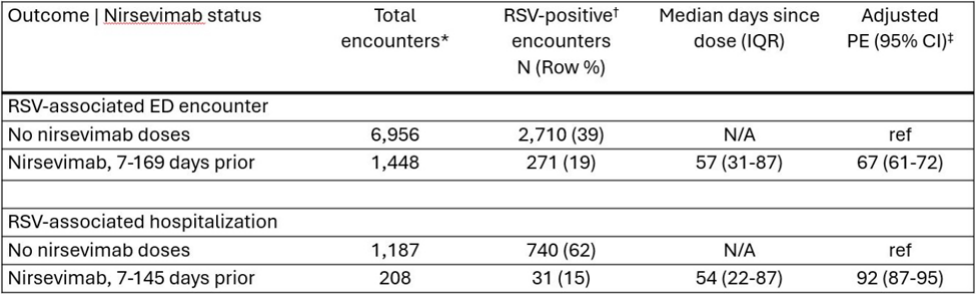
Nirsevimab effectiveness against RSV-associated ED encounters and hospitalization among infants in their first RSV season, VISION, 2023-2024 and 2024-2025 RSV seasons RSV = respiratory syncytial virus; ED = emergency department; IQR = interquartile range; CI = confidence interval; Ref = reference group; PE = product effectiveness; VISION = Virtual SARS-CoV-2, Influenza, and Other respiratory viruses Network *Encounters included those among infants aged <8 months as of October 1 with a diagnosis of RSV-like illness (RLI), excluding infants with evidence of maternal RSV vaccination and infants who received nirsevimab <7 days prior to the index date for the encounter. RLI was defined as ≥1 International Classification of Disease 10th Revision discharge diagnosis code corresponding to one or more of the following: COVID-19 pneumonia, influenza pneumonia, other viral pneumonia, influenza disease, bacterial pneumonia, acute respiratory distress syndrome, asthma exacerbation, respiratory failure, other acute lower respiratory tract infection, sinusitis, acute upper respiratory tract infections, acute respiratory illness signs and symptoms, viral illness not otherwise specified, sepsis, respiratory failure, irritable/fussy infant, respiratory distress of newborn, congenital pneumonia, interstitial emphysema and related conditions, other respiratory conditions originating in the perinatal period, congenital viral diseases, bacterial sepsis of newborn, or other infections specific to the perinatal period. †Encounters with a positive molecular or antigen RSV test 10 days prior to 3 days after the date of the ED encounter or hospital admission were considered RSV-positive. ‡PE was calculated as (1 – adjusted odds ratio) x 100%, with adjusted odds ratio estimated using logistic regression, adjusting for age, race and ethnicity, sex, calendar day, and geographic region.

**Table 2.**
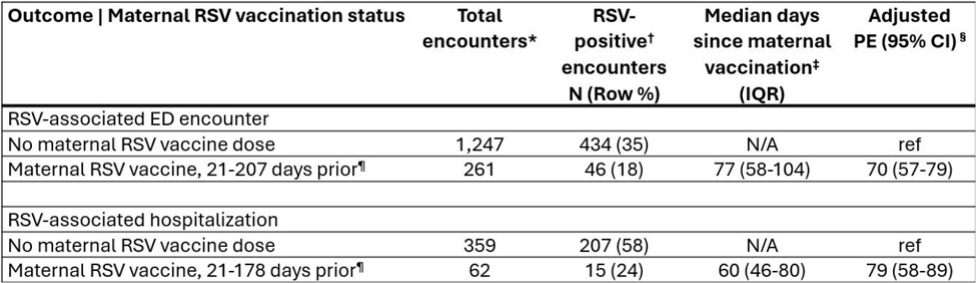
Maternal RSV vaccine product effectiveness against RSV-associated ED encounters and hospitalization among infants in their first RSV season, VISION, 2023-2024 and 2024-2025 RSV seasons RSV = respiratory syncytial virus; ED = emergency department; IQR = interquartile range; CI = confidence interval; Ref = reference group; PE = product effectiveness; VISION = Virtual SARS-CoV-2, Influenza, and Other respiratory viruses Network *Encounters included those among infants in their first RSV season with a diagnosis of RSV-like illness (RLI), excluding infants with evidence of nirsevimab receipt and infants born <14 days after maternal RSV vaccine receipt. RLI was defined as ≥1 International Classification of Disease 10th Revision discharge diagnosis code corresponding to one or more of the following: COVID-19 pneumonia, influenza pneumonia, other viral pneumonia, influenza disease, bacterial pneumonia, acute respiratory distress syndrome, asthma exacerbation, respiratory failure, other acute lower respiratory tract infection, sinusitis, acute upper respiratory tract infections, acute respiratory illness signs and symptoms, viral illness not otherwise specified, sepsis, respiratory failure, irritable/fussy infant, respiratory distress of newborn, congenital pneumonia, interstitial emphysema and related conditions, other respiratory conditions originating in the perinatal period, congenital viral diseases, bacterial sepsis of newborn, or other infections specific to the perinatal period. †Days since maternal vaccination included the number of days between maternal RSV vaccine dose receipt and birth and the number of days since birth. ‡Encounters with a positive molecular or antigen RSV test 10 days prior to 3 days after the date of the ED encounter or hospital admission were considered RSV-positive. §PE was calculated as (1 – adjusted odds ratio) x 100%, with adjusted odds ratio estimated using logistic regression, adjusting for age, race and ethnicity, sex, calendar day, and geographic region. ¶A minimum of 14 days between maternal vaccination and birth was required; infants aged 0-6 days were excluded from this analysis.

**Methods:**

VISION includes integrated health record data with linkages to state and local immunization information systems from 127 EDs and 107 hospitals. We conducted a test-negative analysis using data from 6 VISION sites, including children with ED encounters and hospitalizations with a diagnosis of RSV-like illness (RLI) during October 2023–March 2024 and October 2024–March 2025 among infants in their first RSV season. PE was estimated for each product, comparing the odds of immunization versus no immunization among RSV-positive cases and RSV-negative controls, adjusting for age, race and ethnicity, sex, calendar day, and geographic region.

**Results:**

Of the 8,404 RLI ED encounters and 1,395 RLI hospitalizations among infants in their first RSV season without exposure to maternal RSV vaccine, 2,981 (35%) and 771 (55%) were RSV-positive and 1,448 (17%) and 208 (15%) had evidence of nirsevimab receipt, respectively. Nirsevimab effectiveness was 67% (95% CI: 61-72%) against RSV-associated ED encounters and 92% (95% CI: 87-95%) against RSV-associated hospitalization (Table 1). In 1,508 RLI ED encounters and 421 RLI hospitalizations among infants in their first RSV season without receipt of nirsevimab, 480 (32%) and 222 (53%) were RSV-positive and 261 (17%) and 62 (15%) had evidence of maternal RSV vaccination, respectively. Maternal RSV vaccine PE was 70% (95% CI: 57-79%) against RSV-associated ED encounters and 79% (95% CI: 58-89%) against RSV-associated hospitalization among infants in their first RSV season (Table 2).

**Conclusion:**

Both RSV prevention products were effective in preventing RSV-associated ED encounters and hospitalizations among infants in their first RSV season in the United States. It is important to continue monitoring RSV PE during future RSV seasons.

**Disclosures:**

Steph Battan-Wraith, PhD, Novavax, Inc.: Grant/Research Support Sara Y. Tartof, PhD, MPH, Centers for Disease Control and Prevention: Grant/Research Support Karthik Natarajan, PhD, Centers for Disease Control and Prevention: Grant/Research Support Stephanie Irving, MHS, Westat: Grant/Research Support Shaun J. Grannis, MD, MS, Centers for Disease Control and Prevention: Grant/Research Support|National Institutes of Health NCATS: Grant/Research Support|National Institutes of Health NIMH: Grant/Research Support Sean M. Chickery, DHSc, Centers for Disease Control and Prevention: Grant/Research Support Gabriela Vazquez-Benitez, PhD, MSc, AbbVie: research funding not related to this study|Sanofi: Grant funding for other research not related to this study S. Bianca Salas, MPH, Centers for Disease Control and Prevention: Grant/Research Support|Pfizer: Grant/Research Support Cassandra Bezi, MPH, Centers for Disease Control and Prevention: Grant/Research Support Lina S. Sy, MPH, AstraZeneca: Grant/Research Support|Dynavax: Grant/Research Support|GlaxoSmithKline: Grant/Research Support|Moderna: Grant/Research Support Bruno Lewin, MD, Centers for Disease Control and Prevention: Grant/Research Support|National Institutes of Health: Grant/Research Support William F. Fadel, PhD, Centers for Disease Control and Prevention: Grant/Research Support Sarah W. Ball, MPH, ScD, Centers for Disease Control and Prevention, Contract #200-2019-F-06819: Grant/Research Support|Centers for Disease Control and Prevention, Contract #75D30121D12779: Grant/Research Support|Novavax: Grant/Research Support Jingran Cao, MS, Sanofi Pasteur: Grant/Research Support Charlene E. McEvoy, MD, MPH, Astra Zeneca: Grant/Research Support|Centers for Disease Control and Prevention: Grant/Research Support|Department of Defense: Grant/Research Support|GlaxoSmithKline: Grant/Research Support|National Institutes of Health: Grant/Research Support|PCORI: Grant/Research Support Ryan E. Wiegand, PhD, Merck & Co., Inc.: Stocks/Bonds (Public Company)|Sanofi S.A.: Stocks/Bonds (Public Company)

